# Science Aiding Justice

**Published:** 1861-06

**Authors:** 


### SCIENCE AIDING JUSTICE.

THE facts embodied in the following narration, in connection with a recent murder-trial, show the value of scientific acquirements, and are of exceeding interest to a large class of our readers:

A traveler was found ded in his bed, one morning at a country tavern. His throat was cut at the side, the instrument having pierced the carotid artery; the victim had been for some time wasting away by disease. The landlord was one of the most influential and highly esteemed persons in the neighborhood, was extensively and well connected, and had a large and interesting family. Having been seen very late at night passing through the hall into which the traveler's door opened, the suspicions of certain persons were aroused; and upon being taken into custody, a penknife was found in his pocket, with apparent blood-stains on the large blade, and something similar on the ivory handle. The knife was placed in the hands of an expert physiological chemist for examination. The stain was found to be of blood, and not of iron-rust or paint, as it contained albumen and animal fiber. The blood on the handle contained a large amount of iron, that on the blade comparatively little. As human blood contains ten times as much iron as that of animals, it seemed certain that the knife in question could not have entered a human body; still there was a doubt, because in slow diseases there is a great deficit of iron in the blood, which deficit is a not unfrequent cause of death.

But as the blood on the ivory handle had the full amount of iron for a man in vigorous health, it seemed to show that there were two different kinds of blood, one human certainly, the other possibly so. Hence another mode of inquiry was proposed. The blood of animals and men crystallizes, but in different forms—that of men represented by a perfect square lengthened cube, called prismatic; that of animals, by the cube, tetrahedal, or several-sided hexagonal. This analysis removed the doubts connected with the proceeding, for it demonstrated that the blood on the blade was that of a lower animal, and that on the handle was certainly human.

A third line of investigation was pursued. All the inner surfaces of the human body are covered with a glairy-looking fluid called "mucus," which is differently constituted, according to the part of the body from which it is taken. As observed through a microscope, that which is found about the upper part of the throat presents the appearance of a pavement of bricks or square pieces, hence it is called "tesselated." The mucus from some other parts is conical, looking like a pavement made of round pieces flattened. A third kind, coming from the intestines, seems hairy, ciliated, waving like the tops of long grass under the influence of the wind. Examining the blood on the handle, which was now known to be that of a human being, it was found not to present the pavement-like appearance, but it did clearly show the wavy lines; it could not, therefore, have come from the throat, and as the traveler had no wound except that on the throat, and as the blood on the blade was clearly animal blood and not human, no part of the blood on the knife could have been that of the unfortunate traveler, and therefore the landlord was discharged, when he gave the following statement.

Some days before, while out hunting, he killed several squirrels, and stooped to cut a switch with a knob at the root, on which to string his game; the knife slipped as he cut upwards, and it penetrated the abdomen. In his haste, he wiped the knife clean with some leaves, closed the blade, and in attempting to put it into his pocket it fell on the ground; he picked it up and directed his steps homeward. In a few minutes, one of the squirrels slipped off; he pierced it through with his knife, strung it on the switch, and had not used the knife since. This was plausible, and he showed the wound, not yet entirely healed; but this could easily have been made to answer an object. The physiologist, therefore, proposed, as a mere matter of curious interest, to examine the blood on the blade, and also that on the handle. That on the handle was wavy, ciliary, with the largest amount of iron, showing that it must have been from a man of robust health, and the mucus from the abdomen is always ciliary and never tesselated. Again, the blood adhering to a knife penetrating a living body coagulates—that entering a body already dead never does. The blood on the blade, already shown to be that of a mere animal, was now found to be incoagulable. Hence, that on the blade was shown to be the blood of a mere animal already dead; that on the handle was the blood of a man in vigorous health, and could not have come from the throat, and almost certainly came from the abdomen. When the knife fell on the ground, the handle touched some of the leaves with which it had just been wiped. Thus the chain of evidence for the landlord's innocence was unbroken and perfect. The real culprit was subsequently found, tried, and executed, confessing his guilt.

It is certain that in the progressive march of science and art, the unchangeable laws of nature will be better understood—correcting the errors and fallacies of human judgment; and the testimony of Science will thus aid Justice in forming her opinions and enabling her to give her decisions *with her eyes open.*

---

"Be temperate in all things."—*Bible.*

"He that hateth suretyship is sure."—*Ib.*

"Do justly, love mercy, and walk humbly with thy God."—*Ib.*

"True religion, and undefiled, is this to visit the fatherless and widows in their affliction, and to keep himself unspotted from the world."—*Ib.*

